# *In silico* Identification of 10 Hub Genes and an miRNA–mRNA Regulatory Network in Acute Kawasaki Disease

**DOI:** 10.3389/fgene.2021.585058

**Published:** 2021-03-25

**Authors:** Jin Ma, Huan Gui, Yunjia Tang, Yueyue Ding, Guanghui Qian, Mengjie Yang, Mei Wang, Xiudao Song, Haitao Lv

**Affiliations:** ^1^Department of Pharmacy, Children’s Hospital of Soochow University, Suzhou, China; ^2^Cardiology Department, Children’s Hospital of Soochow University, Suzhou, China; ^3^Institute of Pediatric Research, Children’s Hospital of Soochow University, Suzhou, China; ^4^Clinical Pharmaceutical Laboratory of Traditional Chinese Medicine, Suzhou TCM Hospital Affiliated to Nanjing University of Chinese Medicine, Suzhou, China

**Keywords:** Kawasaki disease, bioinformatical analysis, miRNA–mRNA network, differential expression genes, hub gene

## Abstract

Kawasaki disease (KD) causes acute systemic vasculitis and has unknown etiology. Since the acute stage of KD is the most relevant, the aim of the present study was to identify hub genes in acute KD by bioinformatics analysis. We also aimed at constructing microRNA (miRNA)–messenger RNA (mRNA) regulatory networks associated with acute KD based on previously identified differentially expressed miRNAs (DE-miRNAs). DE-mRNAs in acute KD patients were screened using the mRNA expression profile data of GSE18606 from the Gene Expression Omnibus. The functional and pathway enrichment analysis of DE-mRNAs were performed with the DAVID database. Target genes of DE-miRNAs were predicted using the miRWalk database and their intersection with DE-mRNAs was obtained. From a protein–protein interaction (PPI) network established by the STRING database, Cytoscape software identified hub genes with the two topological analysis methods maximal clique centrality and Degree algorithm to construct a miRNA-hub gene network. A total of 1,063 DE-mRNAs were identified between acute KD and healthy individuals, 472 upregulated and 591 downregulated. The constructed PPI network with these DE-mRNAs identified 38 hub genes mostly enriched in pathways related to systemic lupus erythematosus, alcoholism, viral carcinogenesis, osteoclast differentiation, adipocytokine signaling pathway and tumor necrosis factor signaling pathway. Target genes were predicted for the up-regulated and down-regulated DE-miRNAs, 10,203, and 5,310, respectively. Subsequently, 355, and 130 overlapping target DE-mRNAs were obtained for upregulated and downregulated DE-miRNAs, respectively. PPI networks with these target DE-mRNAs produced 15 hub genes, six down-regulated and nine upregulated hub genes. Among these, ten genes (ATM, MDC1, CD59, CD177, TRPM2, FCAR, TSPAN14, LILRB2, SIRPA, and STAT3) were identified as hub genes in the PPI network of DE-mRNAs. Finally, we constructed the regulatory network of DE-miRNAs and hub genes, which suggested potential modulation of most hub genes by hsa-miR-4443 and hsa-miR-6510-5p. SP1 was predicted to potentially regulate most of DE-miRNAs. In conclusion, several hub genes are associated with acute KD. An miRNA–mRNA regulatory network potentially relevant for acute KD pathogenesis provides new insights into the underlying molecular mechanisms of acute KD. The latter may contribute to the diagnosis and treatment of acute KD.

## Introduction

Kawasaki disease (KD) is responsible for acute systemic vasculitis and it is a high-risk factor of acquired heart disease in children (Gordon et al., 2009). The disease was first reported in 1967 and worldwide incidence has gradually increased in recent years (Uehara and Belay, 2012; Lin and Wu, 2017). Diagnosis of KD still relies on presentation of clinical symptoms, such as persistent fever of more than 5 days, conjunctival non-suppurative hyperemia, red bayberry tongue, rash, lymph node enlargement, fingertip swelling, annular peeling, and systemic vascular inflammatory lesions (McCrindle et al., 2017). Although the etiology of KD remains unknown, the role of alteration of genes and their regulation has become increasingly relevant. The latter is based on the reported presence of susceptibility genes and single nucleotide polymorphisms (Onouchi, 2018; Kumrah et al., 2020) specific signaling pathways (Bijnens et al., 2018; Lv et al., 2019) and genetic predisposition (Uehara et al., 2003). Therefore, diagnosis and treatment of KD will benefit from a comprehensive understanding of the disease at the molecular level.

The most serious complication of KD is coronary artery injury. The latter can develop into coronary artery aneurysm-like changes, coronary artery stenosis, thrombosis, myocardial infarction, and even sudden death (Dietz et al., 2017). At the acute stage, KD-induced vasculitis results in the rapid involvement of inflammatory cells–mainly monocytes, macrophages and activated neutrophils–into the arterial endothelium, which can last for several days (Tsujimoto et al., 2001; Andreozzi et al., 2017). Development of vascular disease and the transition from the acute to the chronic stage correlates with the number of inflammatory cells (Takahashi et al., 2018). In acute KD, vascular endothelial damage may be triggered by inflammatory factors in peripheral blood cells. Thus, exploration of the molecular mechanisms leading to this endothelial vascular inflammation from the perspective of peripheral blood cells should be clinically significant.

In a previous paper, we reported ten differentially expressed miRNAs (DE-miRNAs) from peripheral blood cells of acute KD sufferers and healthy individuals (Chen Y. et al., 2018). miRNAs are endogenous non-coding RNAs that post-transcriptionally reduce gene expression through translational inhibition or mRNA destabilization. MiRNAs are crucial modulators for various cellular biological processes, such as inflammatory response, cell growth or differentiation, which suggests that miRNA–mRNA regulatory networks may play an important role in the pathogenesis of acute KD. Although several miRNAs have been reported to exert functions in the progress of KD (He et al., 2017; Rong et al., 2018), to our knowledge, a systematic and comprehensive analysis of miRNA–mRNA regulatory networks in acute KD is still lacking.

In present study, we have used the mRNA expression profile data of GSE18606 from the Gene Expression Omnibus (GEO) to screen out DE-mRNAs between acute KD and normal control samples. Subsequently, DE-mRNAs commonly appearing as predicted target genes of DE-miRNAs were selected, and hub genes were identified. Finally, integrative miRNA–mRNA regulatory networks associated with acute KD were constructed. Overall, this information should help elucidate the pathogenic mechanism of the acute form of KD, contributing to the early diagnosis and treatment of this disease.

## Materials and Methods

### Microarray Data Source

To obtain the gene expression datasets of acute KD, we searched the GEO database^1^ using the following keywords: “(Kawasaki disease) and “Homo sapiens” [porgn: txid9606]”, and “Expression profiling by array”. After a systematic review, the mRNA expression profile data of GSE profile (GSE18606) was selected and downloaded. GSE18606 was based on GPL6480 (Agilent-014850 Whole Human Genome Microarray 4×44K G4112F). The array data for GSE18606 included nine healthy age-appropriate cases and 20 acute KD cases (8 IVIG non-responding and 12 IVIG-responding cases; Supplementary Table 1). The data were freely available online, and this study did not involve any experiment on humans or animals performed by any of the authors. Ten DE-miRNAs were obtained from our previous study (Chen Y. et al., 2018).

### Differentially Expressed mRNAs Identification

To identify the DE-mRNAs between normal controls and acute KD samples, we used GEO2R–an interactive web tool for comparing two groups of any GEO series. Data processing was performed as described previously (Song et al., 2020). Genes that met the cut-off criteria of adjusted *P*-value (adj. *P*) <0.05 and | log fold change | > 1.0 were considered to be DE-mRNAs. A visual hierarchical cluster analysis was used to show the DEG volcano plot.

### GO Annotation and KEGG Pathway Enrichment Analysis of DE-mRNAs

To obtain function and involved biological processes of DE-mRNAs, Gene Ontology (GO) annotation was performed using the DAVID database, whereas the Kyoto Encyclopedia of Genes and Genomes (KEGG) was used for pathway enrichment analysis. The GO analysis included three categories: biological process (BP), cellular component (CC), and molecular function (MF). *P*-value <0.05 was considered to be statistically significant.

### PPI Network Construction and Hub Genes Identification

The functional protein association networks of DE-miRNAs were obtained using the Search Tool for the Retrieval of Interacting Genes (STRING). DE-mRNAs were first submitted to the STRING database and PPI pairs were extracted with a combined score >0.4. Subsequently, CytoHubba–a plugin in Cytoscape v3.7.2–was used to identify the hub genes using both maximal clique centrality (MCC) and degree methods (Chin et al., 2014). A Venn diagram was used to find the intersecting hub genes.

### Prediction of Target Genes for Identified DE-miRNAs and miRNA–mRNA Construction

miRWalk 3.0 database (Dweep et al., 2011) was used to predict the downstream target genes of identified DE-miRNAs using score ≥0.95 as a cutoff criterion. A Venn diagram web tool was used to obtain candidate genes, from intersecting DE-miRNAs target genes and DE-mRNAs. Construction of an miRNA–mRNA regulation network of these overlapping genes and the corresponding DE-miRNAs was performed with Cytoscape software.

### Prediction of Potential Transcription Factors of DE-miRNAs

Upstream transcription factors of screened DE-miRNAs were predicted using FunRich software 3.1.3–a tool used mainly for functional enrichment and interaction network analysis of genes and proteins (Pathan et al., 2017). The top ten predicted transcription factors were obtained from the input screened upregulated and downregulated DE-miRNAs.

## Results

### Identification of DE-mRNAs

**FIGURE 1:**
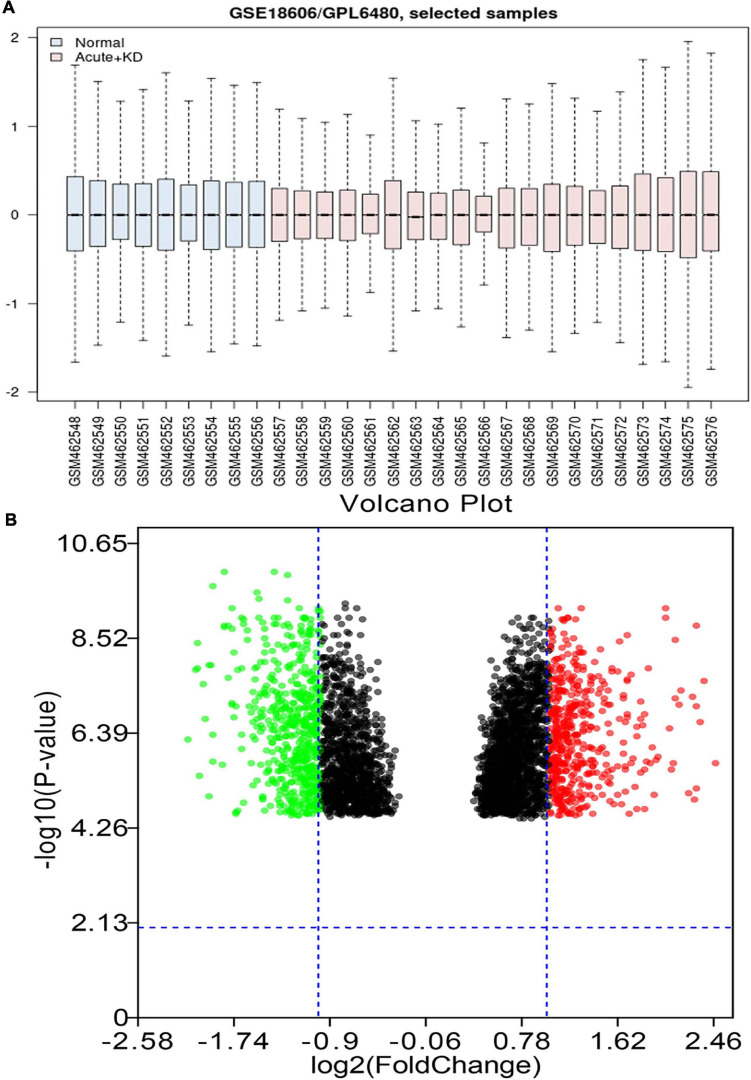
The value distribution of the selected samples, and the volcano plot of the identified DE-mRNAs in GSE18606. **(A)** The blue boxes represent normal samples and the pink boxes represent acute KD cases. Black lines show the median of each data and its distribution represents the standardization degree of the data. **(B)** Volcano plot of the identification of DE-mRNAs. Red, upregulation; green, downregulation.

As shown in Figure 1A, the black lines are almost on the same straight line, which suggests that the standardization level was satisfactory. The 3,731 genes in GSE18606 dataset were plotted, and the red and blue ones represented the up and down-regulation of genes, respectively, as showed in Figure 1B. Among the up and down-regulation of genes, S100A12, and BCL11A were the highest fold change mRNA, respectively. A total of 1,063 DE-mRNAs were identified after GSE18606 dataset analyses, including 472, and 591 up- and down-regulated genes, respectively, which was visualized in Figure 1B and Supplementary Table 2.

### GO Annotation and KEGG Pathway Enrichment Analysis of DE-mRNAs

**FIGURE 2:**
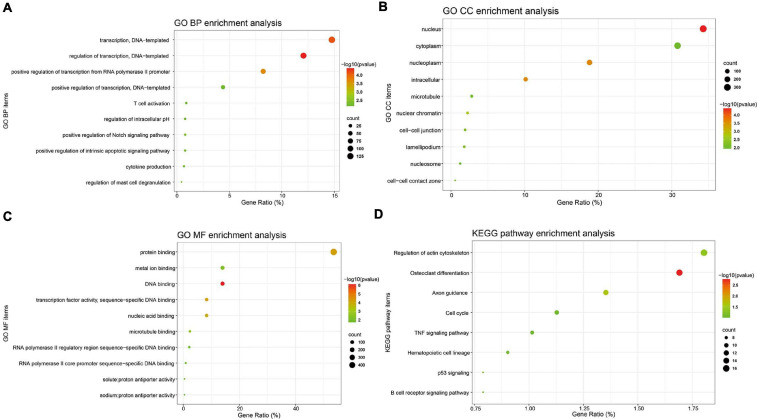
GO annotation and KEGG pathway enrichment analysis of DE-mRNAs. GO functional annotation analysis and KEGG pathway enrichment analysis of these 1,063 DE-mRNAs was performed by the DAVID database. The top 10 enriched GO BP **(A)**, CC **(B)**, and MF **(C)** items, and the enriched KEGG pathways **(D)** were displayed. *Y*-axis: name of GO item or the KEGG signaling pathway; *X*-axis: percentage of the number of genes assigned to a term among the total number of genes annotated in the network; Bubble size, number of genes assigned to a pathway; Color: enriched -log10(*P*-value); Red bubble: indicates a greater significance level.

Gene ontology (GO) BP analysis showed that these 1,063 DE-mRNAs were significantly enriched in various roles (Figure 2A). For GO CC analysis, the top six significantly enriched terms were nucleus, nucleoplasm, intracellular, nuclear chromatin, and cell–cell junction (Figure 2B). The top six significantly enriched MF terms included DNA binding, transcription factor activity (sequence-specific DNA binding), protein binding, nucleic acid binding, microtubule binding, and metal ion binding (Figure 2C). The enriched KEGG items are listed in Figure 2D, including Osteoclast differentiation, Axon guidance, Regulation of actin cytoskeleton, p53 signaling, B cell receptor signaling pathway, Hematopoietic cell lineage, Cell cycle, and TNF signaling pathway. Functional and pathway enrichment analyses of DE-mRNAs were obtained in Supplementary Table 3.

### PPI Network Construction and Hub Genes Identification

**FIGURE 3:**
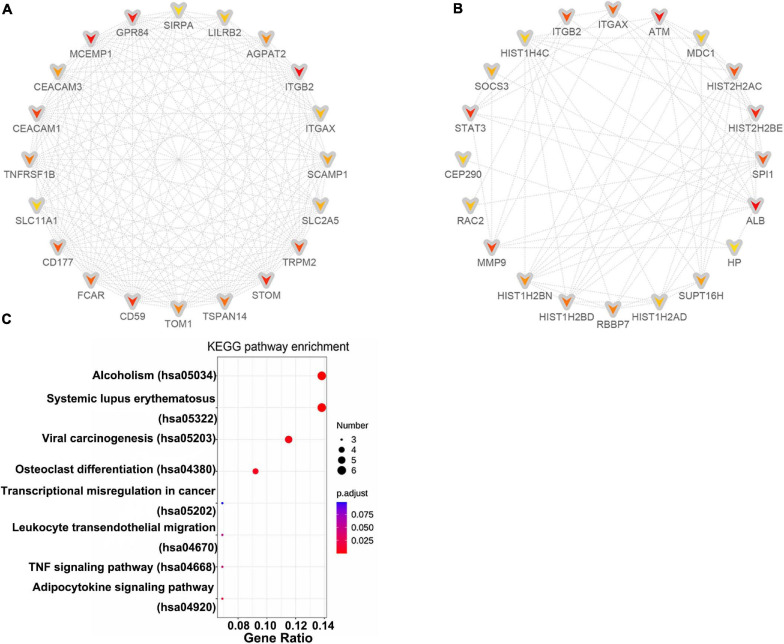
Identification of hub genes. Cytohubba in Cytoscape v3.7.2 was used to find the top 20 hub genes in the PPI network by two analysis methods, namely, MCC **(A)**, and Degree **(B)**. PPI network of the top 20 hub genes was visualized by Cytoscape, and the top 20 hub genes are displayed from red (high MCC/Degree value) to yellow (low MCC/Degree value). **(C)** KEGG enrichment analysis of hub genes.

A PPI network of DE-mRNAs was constructed utilizing Cytoscape software based on the STRING database (Supplementary Figure 1). The network included 985 nodes (genes) and 3,550 edges (interactions), with PPI enrichment *P* value < 1.0E-16. The Cytohubba plugin of Cytoscape was used to rank the top 20 nodes in the PPI network. ITGB2 was the most outstanding gene using the MCC method, followed by MCEMP1, GPR84, STOM, CD59, CEACAM1, TRPM2, CD177, FCAR, TSPAN14, TNFRSF1B, TOM1, AGPAT2, CEACAM3, SCAMP1, SLC2A5, ITGAX, LILRB2, SLC11A1, and SIRPA (Figure 3A). ALB has the highest connectivity degree (75), followed by ATM (55), HIST2H2BE (53), STAT3 (51), MMP9 (47), SPI1 (45), ITGB2 (45), HIST2H2AC (45), ITGAX (40), HIST1H2BD (38), RBBP7 (37), HIST1H2BN (35), SUPT16H (34), SOCS3 (33), HIST1H2AD (32), RAC2 (32), CEP290 (31), HIST1H4C (31), MDC1 (31), and HP (30) (Figure 3B). Pathway enrichment analysis of these hub genes suggested involvement in pathway regulation, including Systemic lupus erythematosus, Alcoholism, Viral carcinogenesis, Osteoclast differentiation, Adipocytokine signaling pathway, and TNF signaling pathway (Figure 3C).

### Prediction of DE-miRNAs Downstream Target Genes

We previously have identified ten DE-miRNAs between acute KD and normal control, including seven up-regulated DE-miRNAs (hsa-let-7b-5p, hsa-miR-223-3p, hsa-miR-765, hsa-miR-4485-3p, hsa-miR-4644, hsa-miR-4800-5p, and hsa-miR-6510-5p) and three down-regulated DE-miRNAs (hsa-miR-33b-3p, hsa-miR-4443, and hsa-miR-4515) (Chen Y. et al., 2018). The predicted downstream target genes for the ten DE-miRNAs were 10,203 for the up-regulated and 5,310 for the down-regulated DE-miRNAs (see Table 1 and Supplementary Table 4).

**TABLE 1:** The target gene count for each DE-miRNA.

**miRNA ID**	**Target gene count**
**Upregulation**	
hsa-let-7b-5p	2815
hsa-miR-223-3p	654
hsa-miR-765	4393
hsa-miR-4485-3p	1176
hsa-miR-4644	2225
hsa-miR-4800-5p	2872
hsa-miR-6510-5p	5286
**Downregulation**	
hsa-miR-33b-3p	1977
hsa-miR-4443	2710
hsa-miR-4515	1967

**FIGURE 4:**
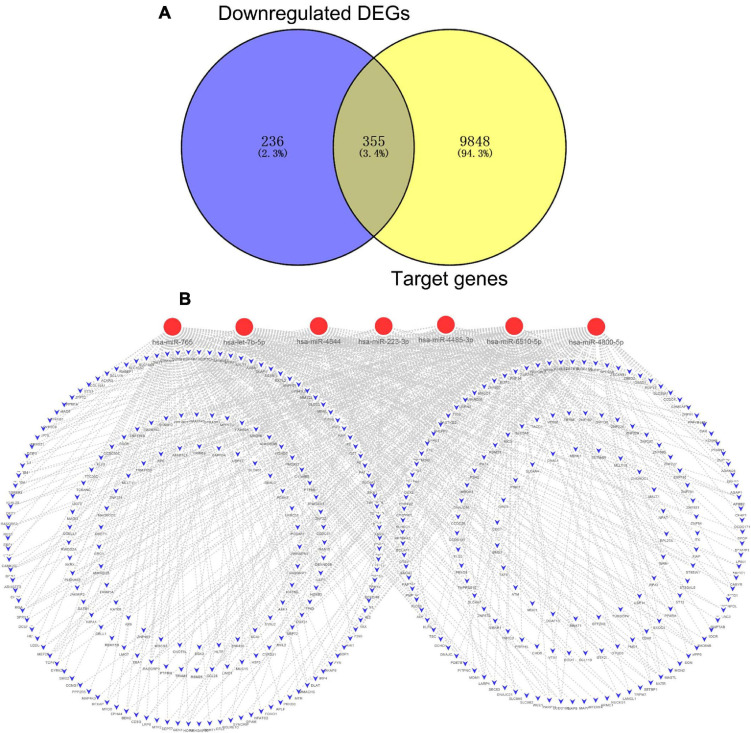
The regulatory network of upregulated DE-miRNAs and candidate downregulated DE-mRNAs in acute Kawasaki disease. **(A)** Screen of candidate genes. The intersection of target genes of upregulated DE-miRNAs and downregulated DE-mRNAs. **(B)** The construction of regulatory network of upregulated DE-miRNAs and candidate genes. Red, upregulation; blue, downregulation.

**FIGURE 5:**
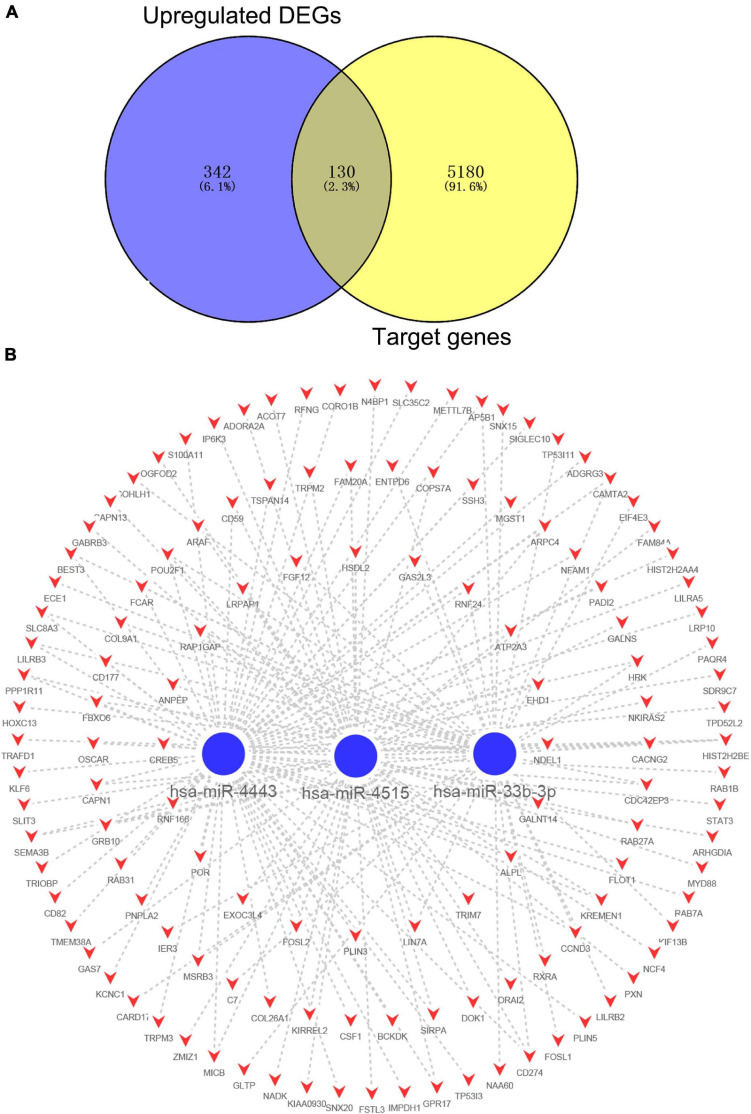
The regulatory network of downregulated DE-miRNAs and candidate upregulated DE-mRNAs in acute Kawasaki disease. **(A)** Screen of candidate genes. The intersection of target genes of the intersection of downregulated DE-miRNAs and upregulated DE-mRNAs. **(B)** The construction of regulatory network of downregulated DE-miRNAs and candidate genes. Red, upregulation; blue, downregulation.

After combining the analysis of DE-mRNAs and target genes of DE-miRNAs (Supplementary Table 5), we further screened 355 candidate target genes for upregulated (Figure 4A) and 130 candidate target genes for downregulated DE-miRNAs (Figure 5A). Subsequently, the candidate miRNA–mRNA regulatory network associated with the development of acute KD was constructed (Figures 4B, 5B and Supplementary Table 6).

### Further Analysis of the Overlapping Genes for the Target Genes of DE-miRNAs and DE-mRNAs

**FIGURE 6:**
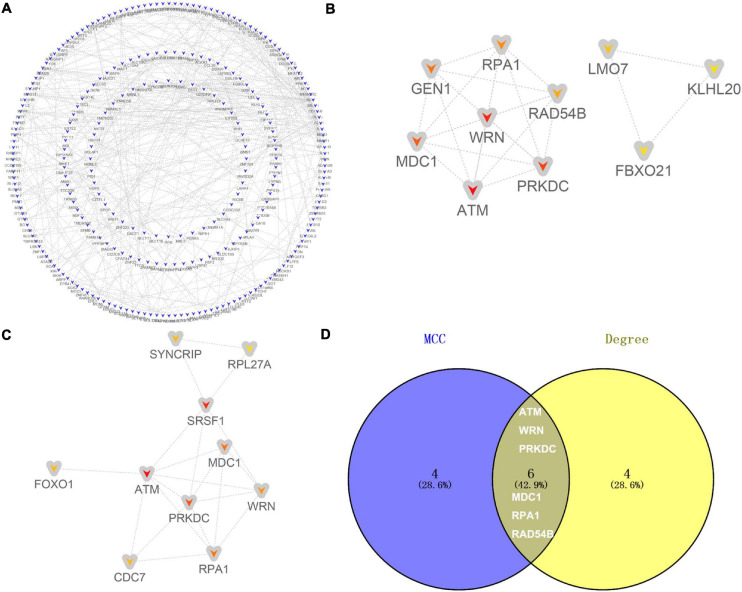
Identification of hub genes for the candidate target genes corresponding to upregulated DE-miRNAs. **(A)** PPI network constructed with all the 355 candidate target genes of upregulated DE-miRNAs were performed using the STRING. The nodes represent proteins, and the edges represent the interaction of the proteins. Blue, downregulation. Cytohubba in Cytoscape was used to find the top 10 hub genes in the PPI network by two analysis methods, namely, MCC **(B)**, and Degree **(C)**. PPI network of the top 10 hub genes was visualized by Cytoscape, and the top 10 hub genes are displayed from red (high MCC/Degree value) to yellow (low MCC/Degree value). **(D)** The overlapping hub genes in top 10 by the two topological methods.

The 485 candidate target genes corresponding to upregulated (355) and downregulated (130) DE-miRNAs were submitted to the STRING database. For the 355 candidate target genes of upregulated DE-miRNAs, a network with 355 nodes (genes) and 454 edges (interactions) (PPI enrichment *P* value < 5.73E-10) was obtained (Figure 6A and Supplementary Table 6). The top 10 hub genes were shown according to MCC (Figure 6B) and Degree (Figure 6C) methods. Six overlapping hub genes were obtained: ATM, WRN, PRKDC, MDC1, RPA1, and RAD54B (Figure 6D).

**FIGURE 7:**
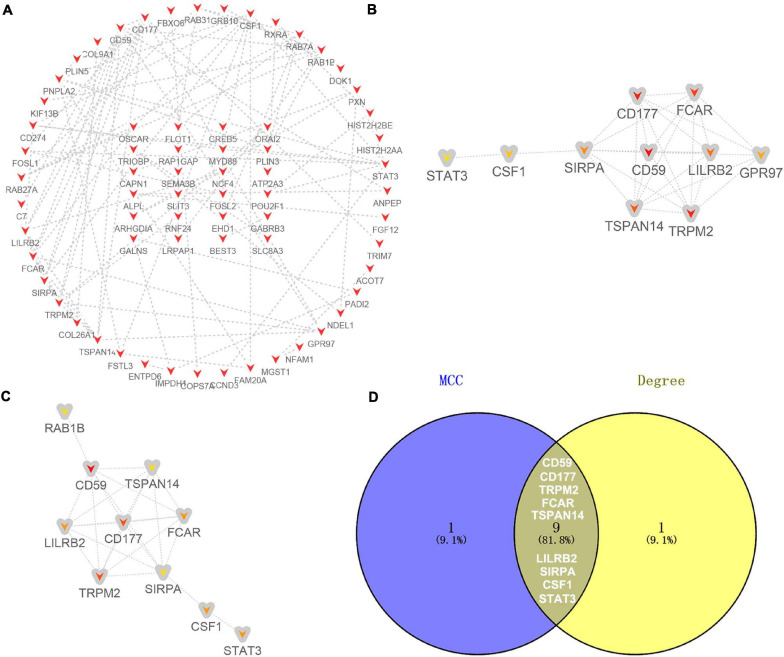
Identification of hub genes for the candidate target genes corresponding to downregulated DE-miRNAs. **(A)** PPI network constructed with all the 130 candidate target genes of upregulated DE-miRNAs were performed using the STRING. The nodes represent proteins, and the edges represent the interaction of the proteins. Red, upregulation. Cytohubba in Cytoscape was used to find the top 10 hub genes in the PPI network by two analysis methods, namely, MCC **(B)**, and Degree **(C)**. PPI network of the top 10 hub genes was visualized by Cytoscape, and the top 10 hub genes are displayed from red (high MCC/Degree value) to yellow (low MCC/Degree value). **(D)** The overlapping hub genes in top 10 by the two topological methods.

For the 130 candidate target genes of downregulated DE-miRNAs, a network with 130 nodes (genes) and 99 edges (interactions) (PPI enrichment *P* value <1.61E-05) was obtained (Figure 7A and Supplementary Table 6). The hub genes were obtained according to MCC (Figure 7B) and Degree (Figure 7C) methods. Nine overlapping hub genes were obtained: CD59, CD177, TRPM2, FCAR, TSPAN14, LILRB2, SIRPA, CSF1, and STAT3 (Figure 7D).

### Identification of the miRNA-Hub Gene Regulatory Network in Acute Kawasaki Disease

**FIGURE 8:**
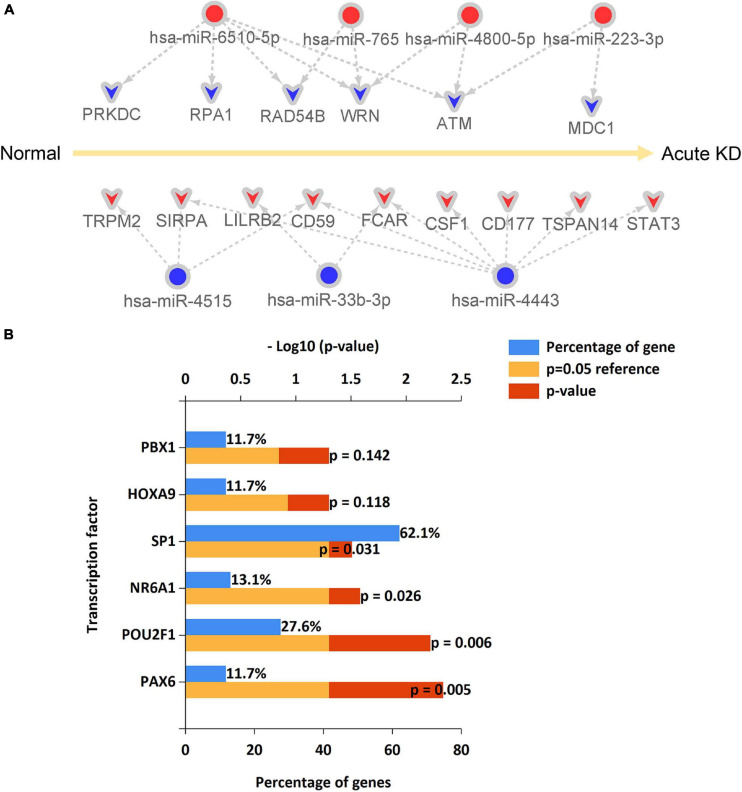
An miRNA–hub gene regulatory network in acute Kawasaki disease construction and predicted transcription factors of DE-miRNAs. **(A)** An miRNA–hub gene regulatory network was visualized by Cytoscape. Red, upregulation; blue, downregulation. **(B)** The upstream transcription factors of DE-miRNAs were predicted by using FunRich software.

From the predicted miRNA–mRNA pairs, the miRNA-hub gene regulatory network associated with the development of acute KD was constructed (Figure 8A and Supplementary Table 6) resulting in seven DE-miRNAs. The upstream transcription factors of these seven DE-miRNAs, including hsa-miR-6510-5p, hsa-miR-765, hsa-miR-4800-5p, hsa-miR-223-3p, hsa-miR-4515, hsa-miR-33b-3p, and hsa-miR-4443, were further predicted by using FunRich software. The top 10 transcription factors for these seven DE-miRNAs are shown in Figure 8B. The present study identified the four significant transcription factors for these seven DE-miRNAs. These included transcription factor PAX6, POU2F1, NR6A1, and SP1.

## Discussion

Children suffering acute stage KD experience outbreaks of inflammatory factors in peripheral blood, and also inflammatory changes of small and medium-sized blood vessels in the whole body. In the clinic, specific inhibitors of tumor necrosis factor (TNF) infliximab and enalapril have been used to treat children with intravenous immunoglobulin (IVIG)-insensitive KD (Tremoulet et al., 2014; Portman et al., 2019). Cyclosporine, an inhibitor of the calmodulin in the NFAT signaling pathway, is an alternative treatment for children with refractory KD and IVIG-unresponsive KD (Aoyagi et al., 2015). Despite these treatments, the role of gene alteration in the pathogenesis of KD remains to be elucidated. Recently, microarray technology coupled with bioinformatics tools has been used to identify the novel genes associated with the pathogenesis, diagnosis and treatment of KD (Wu et al., 2019; Yazdan et al., 2020). Herein, we have used mRNA expression profile data of GSE18606 to identify hub genes in whole blood that are associated with acute KD. KEGG pathways enriched by these hub genes included systemic lupus erythematosus, alcoholism (Diniz et al., 2012), osteoclast differentiation (Chang et al., 2019), and TNF signaling pathway have been reported to be associated with KD. Using the screened DE-miRNAs from our previous study (Chen Y. et al., 2018), an miRNA–mRNA regulatory network was constructed which is potentially relevant in the pathogenesis of acute KD.

At the current study, 38 hub genes were identified in the PPI network of DE-mRNAs through using MCC and degree methods. Among these hub genes, CEACAM1, SLC11A1, and MMP9 were reported to be associated with KD etiology. CEACAM1 was identified to be upregulated in acute KD at the current study. In agreement with our results, higher CEACAM1 expression levels were associated with KD etiology including an increased percentage of unsegmented neutrophils, fewer days of illness, and higher levels of C-reactive protein (Popper et al., 2007). Allele 1 of the (GT) n repeat sequence of the 5′ promoter of SLC11A1 is highly expressed in KD patients and the gene has weak promoter activity, which explains the possible infectious etiology of KD and the possible genetic risk in the Japanese population (Ouchi et al., 2003). In the present study, we also found that SLC11A1 was upregulated in plasma of acute KD patients. MMP9 is an independent risk factor for coronary artery disease in KD (Korematsu et al., 2012), and the MMP-9 mRNA level was increased in KD patients with coronary artery disease and decreased at least 3 weeks after IVIG treatment (Kuo et al., 2017). In the heart of KD mouse models, MMP-9 was reported to be up-regulated (Shangguan et al., 2014). Thus, MMP9 may be related to the etiology of coronary artery disease in KD. In acute KD, the MMP-9 mRNA expression was shown to be upregulated at the present study, which suggests that MMP-9 may also be related to the KD etiology. In addition, several evidences have documented the upregulation of S100A12 in plasma of acute KD patients (Foell et al., 2003; Ye et al., 2004; Wittkowski et al., 2007), which has been identified to be upregulated with the highest fold change mRNA at the current study. Furthermore, S100A12 become decreased after gammaglobulin treatment (Foell et al., 2003). S100A12 was reported to induce sterile inflammatory activation of HCAECs in an IL-1β-dependent manner, suggesting the role of S100A12 in the pathogenesis of KD (Armaroli et al., 2019). Thus, S100A12 could serve as a novel target for future therapeutic interventions in KD. At the present study, the downregulation of BCL11A with the highest fold change mRNA was identified in acute KD. The BCL11A expression level in KD has not been reported in the literature so far. Thus, the role of BCL11A in KD deserves to be further studied.

We obtained 355, and 130 overlapping target DE-mRNAs for upregulated and downregulated DE-miRNAs, respectively. PPI networks with these overlapping target DE-mRNAs identified 15 hub genes. Out of these, ten were identified as hub genes in the PPI network of DE-mRNAs, and included two downregulated (ATM, and MDC1) and eight upregulated (CD59, CD177, TRPM2, FCAR, TSPAN14, LILRB2, SIRPA, and STAT3) DE-mRNAs. Four of these (CD177, FCAR, STAT3, and CD59) have been found to be linked to KD.

CD177 is an adhesion molecule that binds to CD31 on the surface of endothelial cells, platelets and leukocytes, and participates in the adhesion and migration of neutrophils to endothelial cells (Pliyev and Menshikov, 2012). Upregulation of CD177 in plasma of acute KD patients is supported by high abundance of CD177 transcript (Ko et al., 2019). A case-control study also found that CD177 was up-regulated in KD patients, whereas levels decreased after IVIG treatment (Huang et al., 2019). High expression of CD177 may also be related to IVIG insensitivity (Huang et al., 2019). Higher FCAR mRNA levels in KD patients have also been reported (Chang et al., 2021). FCAR plays an important role in IgA-mediated immune regulation and effector function. FCAR receptor binds to immunoglobulin (Ig) A to mediate mucosal immunity in asthma (Jasek et al., 2004). STAT3 has been widely studied in tumors, where an abnormal STAT3 signaling pathway induces inflammation and immunosuppression (Yu et al., 2014). STAT3 was activated in T cells of child KD patients (Qi et al., 2017) and Candida albicans water-soluble fraction-induced the mouse KD model (Suzuki et al., 2017). Our previous work confirmed that miR-223 can inhibit the activation of the STAT3 signal pathway by targeting the inhibition of IL6ST, thus inducing vascular endothelial cell injury, suggesting that STAT3 may play a role in endothelial vascular inflammatory injury in KD (Wang et al., 2019). CD59, a complement regulatory protein, maintains the integrity of blood vessels by protecting endothelial cells from injury in local inflammatory sites. However, CD59 levels in plasma were found to be low at the early stage of KD (Song et al., 2016). In agreement with our results, plasma CD59 concentrations increased in acute-phase KD patients compared with subacute-phase KD patients (Zou et al., 2015). Therefore, this inconsistent result requires further investigation.

The remaining six hub genes, i.e., two downregulated DE-mRNAs (ATM, and MDC1) and four upregulated DE-mRNAs (TRPM2, TSPAN14, LILRB2, and SIRPA) have not been directly linked to KD. However, they are likely to be involved in vascular endothelial function, inflammatory response or immune regulation. ATM, SIRPA, and LILRB2 are potential targets in tumor immunotherapy (Chen H. M. et al., 2018; Logtenberg et al., 2019; Zhang et al., 2019). In particular, there are many unanswered questions in the role of the SIRPA-CD47 signal pathway in autoimmune diseases. MDC1, a nuclear mediator/junction protein, plays an important role in mediating DNA damage (Jungmichel and Stucki, 2010). TRPM2 is an M2 transient receptor potential located on vascular endothelium, and its high expression in endothelial cells plays an important role in immunity, endothelial barrier and endothelial cell apoptosis (Yamamoto et al., 2008; Wang et al., 2016). TSPAN14 is a member of the TspanC8 subgroup, and its interaction with ADAM10 is important in embryonic development and related to inflammatory diseases (Noy et al., 2016). Thus, these six hub genes may be potentially linked to KD.

MicroRNA (miRNA) participates in a variety of biological processes through post-transcriptional regulation. In our previous work, we analyzed the expression profile of miRNA in peripheral blood of acute KD and screened out ten DE-miRNAs (Chen Y. et al., 2018). From that study, we predicted target genes of DE-miRNAs, and in combination with the DE-mRNAs obtained from data mining we have constructed an miRNA–mRNA regulatory network that will help in the diagnosis or treatment of acute KD. In this network, hsa-miR-4443 may play an important role in KD since it had the highest connectivity with DE-mRNAs, seven of which are upregulated (SIRPA, CD59, FCAR, CD177, TSPAN14, and STAT3), although no studies on this potential direct regulatory action have been performed so far. It has reported that miR-4443 is involved in T cell-mediated immune response (Shefler et al., 2018) that is associated with the pathogenesis of KD (Galeotti et al., 2016), suggesting that miR-4443 may play a role in immune regulation in KD. Five downregulated DE-mRNAs may be candidate target genes of hsa-miR-6510-5p, and one of them is a hub gene ATM. However, no relevant reports were found between miR-6510-5p and vascular inflammation or immune response. Two downregulated DE-mRNAs, ATM, and MDC1, are possibly negatively targeted by hsa-miR-223-3p. The latter has been verified by RT-PCR to be upregulated in our previous study (Chen Y. et al., 2018). Together with other studies (Chu et al., 2017; Parra-Izquierdo et al., 2020; Zhang et al., 2020), our recent find (Wang et al., 2019) supported that miR-223 is an important regulator of vascular endothelial cell injury in KD. Thus miR-223 may be a novel target for the treatment of KD. In our previous study, hsa-miR-765 has been identified to be upregulated in acute KD (Chen Y. et al., 2018). It has reported that the level of circulating miR-765 in patients with coronary heart disease is upregulated (Infante et al., 2019). Since KD is one of the important risk factors of acquired heart disease in children, miR-765 may be a pathogenetic mechanism linking KD with the development of acquired heart disease. The role of the remaining six DE-miRNAs we previously identified including hsa-let-7b-5p, hsa-miR-4485-3p, hsa-miR-4644, hsa-miR-4800-5p, hsa-miR-33b-3p, and hsa-miR-4515 in KD have not been reported in the literature so far. More work is needed to further explore the role of these identified DE-miRNAs in KD.

In summary, our *in silico* analysis has identified hub genes associated with acute KD. Among these hub genes, two downregulated DE-mRNAs (ATM, and MDC1) and four upregulated DE-mRNAs (TRPM2, TSPAN14, LILRB2, and SIRPA) have been linked firstly to KD. The present study, for the first, constructed an miRNA–mRNA regulatory network associated with acute KD, which provides new insights into the molecular mechanisms of acute KD, contributing to its diagnosis and treatment. However, we point out that the screened DE-miRNA and DE-mRNA came from different clinical samples, which limits the analysis. Only one GEO dataset was chosen at the current study, and we understand that this represents one more limitation of the current study. Also, the miRNA target genes were predicted by the software database, and its relevance has not been confirmed experimentally. Finally, the core genes predicted in our miRNA–mRNA network must be verified by a large number of clinical KD samples. This is ongoing work in our lab.

## Data Availability Statement

The datasets presented in this study can be found in online repositories. The names of the repository/repositories and accession number(s) can be found in the article/Supplementary Materials.

## Author Contributions

HL and XS conceived and designed the experiments. JM, XS, HG, YT, YD, GQ, MY, and MW acquired and analyzed the data. JM, XS, and HG drafted the manuscript. HL was responsible for the integrity of the work as a whole. All authors read and approved the final manuscript.

## Conflict of Interest

The authors declare that the research was conducted in the absence of any commercial or financial relationships that could be construed as a potential conflict of interest.

## Footnotes

1 https://www.ncbi.nlm.nih.gov/geo/

## Supplementary Material

Supplementary Figure 1 PPI network of DE-mRNAs. PPI network constructed with all the 1063 DE-mRNAs were performed using the STRING. The nodes represent proteins, and the edges represent the interaction of the proteins. Red, upregulation; blue, downregulation.

Supplementary Table 1 Annotation of 29 samples from GSE18606.

Supplementary Table 2 Significantly differentially expressed mRNA in GSE18606.

Supplementary Table 3 GO annotation and KEGG pathway enrichment analysis of DE-mRNAs.

Supplementary Table 4 Prediction of DE-miRNAs downstream target genes.

Supplementary Table 5 355 and 130 overlapping target DE-mRNAs.

Supplementary Table 6 Some project files to construct the miRNA–mRNA regulatory network.
