# Supplementary figures and images for: In silico Identification of 10 Hub Genes and an miRNA–mRNA Regulatory Network in Acute Kawasaki Disease
Source: Front Genet. 2021 Mar 25;12:585058. doi: 10.3389/fgene.2021.585058 (PMC8044791; doi:10.3389/fgene.2021.585058)

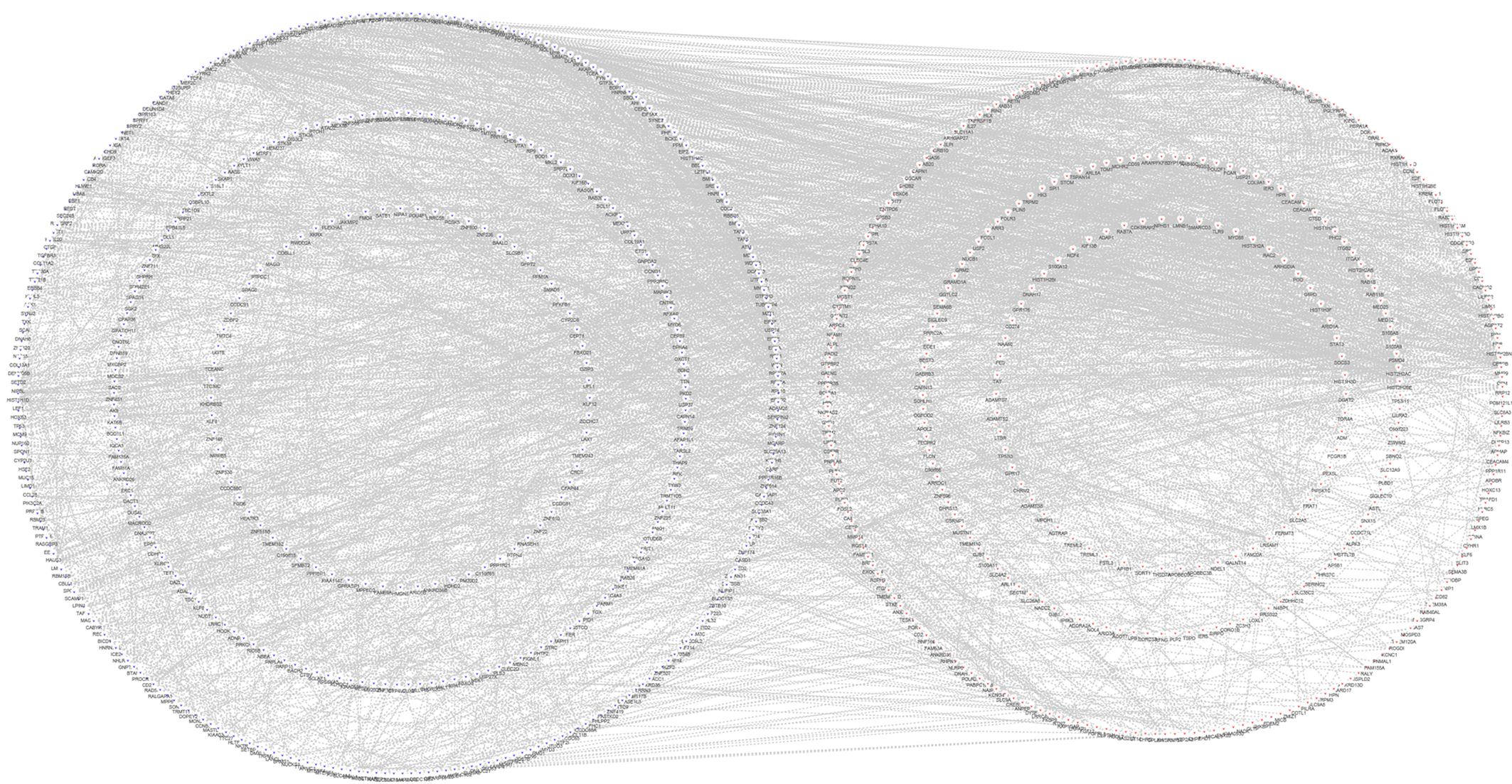

Supplement: Supplementary Figure 1 — PPI network of DE-mRNAs. PPI network constructed with all the 1063 DE-mRNAs were performed using the STRING. The nodes represent proteins, and the edges represent the interaction of the proteins. Red, upregulation; blue, downregulation. [file Image_1.TIF]
